# Reply to Reimann et al.

**DOI:** 10.1038/s41379-018-0197-1

**Published:** 2019-01-21

**Authors:** Loren E. Clarke, Sancy A. Leachman

**Affiliations:** 1 Myriad Genetics, Inc., Salt Lake City, UT USA; 2 Department of Pathology, Oregon Health & Science University, Portland, OR USA; 3 Melanoma Research Program, Knight Cancer Institute, Portland, OR USA

**Keywords:** Diagnostic markers, Melanoma, Cancer screening

We read with interest the recent report by Reimann et al. [1], in which authors from Inform Diagnostics™ compare their histopathologic diagnoses of melanocytic neoplasms to the results of single-nucleotide polymorphism (SNP) array (performed at Memorial Sloan-Kettering Cancer Center, New York City, NY), melanoma fluorescence in situ hybridization (FISH) (performed by the authors at Inform Diagnostics, Irving, TX), and the myPath Melanoma test (Myriad Genetic Laboratories, Salt Lake City, UT). We submit that several methodological weaknesses compromise the validity of the study’s results and conclusions, including (1) use of a suboptimal reference standard and (2) adoption of an alternative, author-devised classification system that is not consistent with the clinically-validated classification system provided with the myPath test results.

The scarcity of melanocytic neoplasms with known outcomes often necessitates use of histopathology-based reference standards, but such standards are limited by the inherent inter-observer variability and diagnostic discordance that occurs even among experts [2–6]. The diagnoses for 70% (187/268) of the Reimann et al. [1] cohort were assigned by just a few individuals representing a single commercial laboratory (Inform Diagnostics™) and therefore might reflect the particular diagnostic predisposition of this group. This, in combination with the tendency for melanoma ‘over-diagnosis’ noted by the authors themselves and many others [7–9], may have contributed to the apparent low sensitivity of the myPath test observed here (50–72%) vs. that observed previously (92%) in a larger cohort (*n* = 736) where diagnoses were assigned by a panel of dermatopathologists from a variety of different institutions [10]. When subjective reference standards such as diagnosis by histopathology generate two very different estimates of a test’s performance, an objective standard such as clinical outcome may help determine which estimate is more accurate. There is no objective standard for non-metastasizing melanocytic neoplasms, but in a large cohort of cases proven to be malignant melanomas by the development of distant metastases, the sensitivity of the myPath test was 94% (Table 3) [11].

The authors’ conclusions are also influenced by their decision to re-classify all cases with indeterminate myPath results as ‘benign’ in their calculations of sensitivity and specificity. The authors claim this was done to 'avoid exclusion of indeterminate results in statistical analysis,' yet their own indeterminate results are, in fact, excluded from statistical analyses. Cases that they declared ‘diagnostically ambiguous’ are eliminated from some calculations (see Table 3 in Reimann et al. [1]), and lesions for which they cannot reach consensus diagnoses are excluded from others (see Table 5 in Reimann et al. [1]). In addition, the authors classify indeterminate myPath results differently from one calculation to the next. Specifically, they are considered negative when assessing sensitivity and specificity and when comparing myPath to FISH interpretation, but for agreement calculations the ‘indeterminate’ designation is reinstated and they are counted as discordant. Interestingly, in these agreement calculations, re-assigning myPath indeterminate results to *either* category—positive *or* negative—would have substantially improved concordance. In fact, in every circumstance, the classification strategy selected by Reimann et al. [1] is the one that produces the least favorable estimate of myPath performance. In clinical use, indeterminate myPath results are not dismissed as benign; on the contrary, they are considered carefully and on a case-by-case basis within the context of clinical findings, histopathologic features, and all other relevant data. The re-interpretation of indeterminate results as benign in the clinical setting represents a potential danger to tested patients and we strongly discourage this practice, as do several publications cited by the authors themselves [12, 13].

**Fig. 1:**
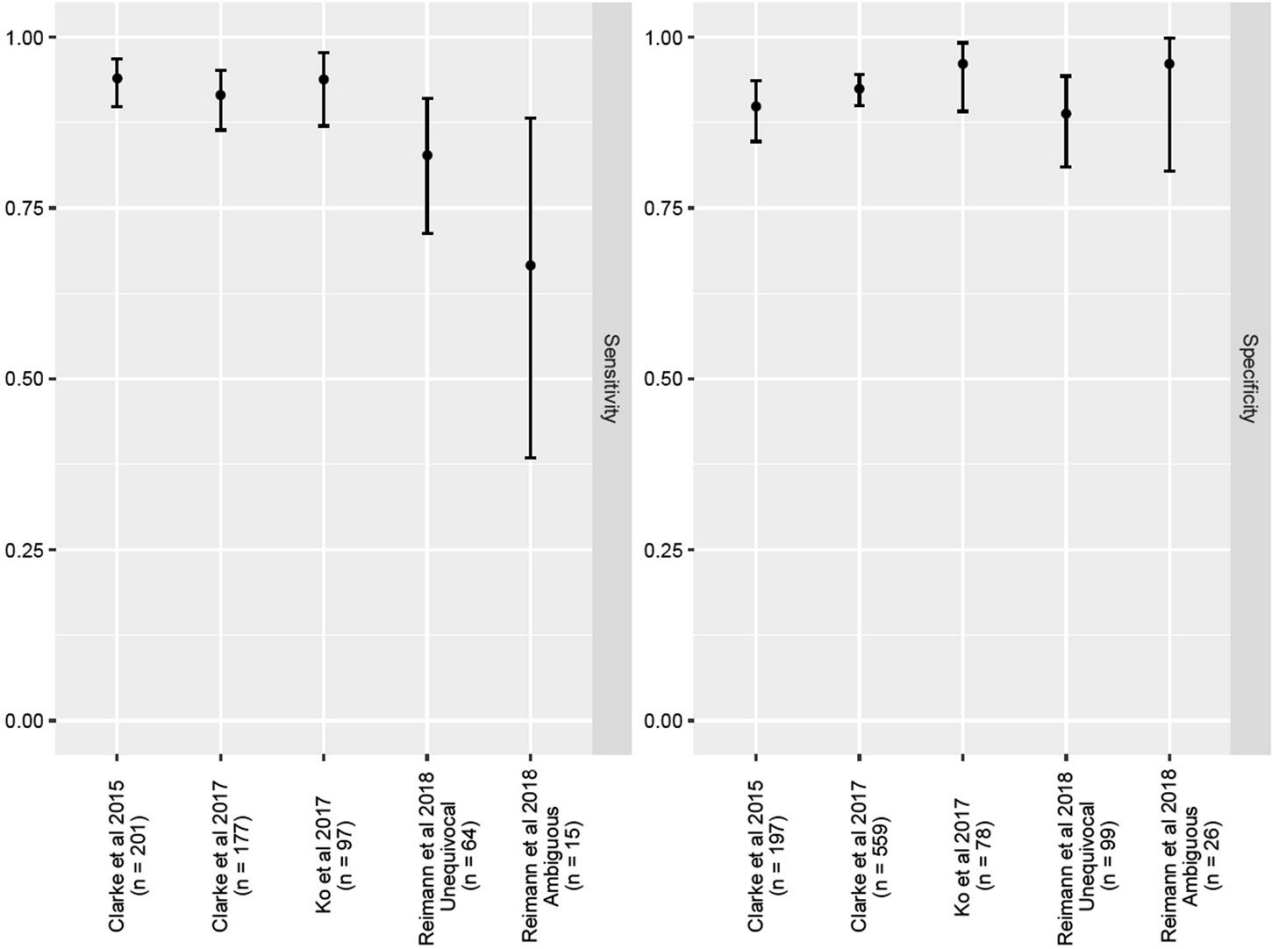
Sensitivity and specificity with confidence intervals

Re-analysis of the data generated by Reimann et al. [1] using the clinically-validated myPath system improves every calculation of the test’s performance (Tables 1–3), and while sensitivity remains lower than that reported in the clinical validation studies, the small number of cases studied by Reimann et al. [1] produce wide confidence intervals that make such comparisons questionable (Fig. 1). It is notable that, when calculated according to the validated classification system, the myPath test’s sensitivity and specificity for cases considered 'unequivocal' and 'ambiguous' by Reimann et al. [1] do not differ, supporting prior observations that performance is consistent regardless of whether lesions are deemed 'ambiguous' or 'straightforward' (Table 3 and Fig. 1) [11, 14]. This suggests that the negative/benign myPath results for 5 of the 15 'ambiguous' melanomas may not reflect a low test sensitivity, but rather a tendency for 'over-classification of benign lesions with atypical features' noted by the authors themselves.

**Table 1:** Comparison of myPath test performance by the Reimann et al. [1] classification system vs. the clinically-validated classification system provided with the myPath test results

Case type	Reference standard	Analysis	Reported by Reimann et al. [1]	Using validated myPath test results
Unequivocal	Histopathologic diagnosis	Sensitivity	72%	83%
		Specificity	89%	89%
Ambiguous	Histopathologic diagnosis	Sensitivity	50%	67%
		Specificity	96%	96%

**Table 2:** Comparison of myPath and FISH in Reimann et al. [1] using the clinically-validated classification system provided with the myPath test results

Case type	Sensitivity	Specificity
All ambiguous cases		
FISH	61%	100%
myPath	67%	96%
‘Challenging spitzoid cases’		
FISH	60%	100%
myPath	75%	95%
‘Challenging nevoid cases’		
FISH	63%	100%
myPath	80%	100%

**Table 3:** Sensitivity and specificity (with confidence intervals) observed in Reimann et al. [1] and prior studies of myPath melanoma (using clinically-validated classification system)

Study	Reference standard	Sensitivity	95% Confidence interval	Specificity	95% Confidence interval
Clarke et al. [14]	Histopathology	94%	90%, 97%	90%	85, 93%
Clarke et al. [10]	Histopathology	92%	86%, 95%	93%	90%, 95%
Reimann et al. [1]	Histopathology				
'Unequivocal'		83%	71%, 91%	89%	81%, 94%
'Ambiguous'		67%	38%, 88%	96%	80%, 100%
Ko et al. [11]	Clinical outcome	94%	87%, 98%	96%	89%, 99%

The accuracy of the myPath melanoma test has been evaluated in three separate peer-reviewed clinical validation studies [10, 11, 14] and two clinical utility studies [15, 16] performed in collaboration with more than 16 dermatopathologists from 10 academic institutions. We believe the preponderance of the data demonstrate the myPath melanoma test to be a useful ancillary diagnostic method. However, no test is perfect, nor is any study without flaw, and knowledge of the molecular pathogenesis of specific subtypes and melanocytic neoplasms in general continues to evolve. All diagnostic methods for melanocytic neoplasms, including histopathology, warrant continued investigation.
